# Variation of the cephalic and basilic veins: A case report

**DOI:** 10.15171/jcvtr.2017.40

**Published:** 2017-10-15

**Authors:** Akram Sadeghi, Mohsen Setayesh mehr, Ebrahim Esfandiari, Shabnam Mohammadi, Hamid Baharmian

**Affiliations:** ^1^Department of Anatomy, School of Medcine, Birjand University of Medical Sciences, Birjand, Iran; ^2^Department of Anatomy, School of Medcine, Isfahan University of Medical Sciences, Isfahan, Iran; ^3^Neurogenic Inflamination Research Center, Mashhad University of Medical Sciences, Mashhad, Iran

**Keywords:** Variation, Vein, Human

## Abstract

Cephalic and basilic veins begin their path from around the wrist and continue towards the area above the forearm. The basilic vein becomes deep around the mid-arm, while the cephalic vein becomes deep around the upper forearm, in deltopectoral groove. The superficial veins are most commonly used for vein puncture, transfusion, bypass graft, and cardiac catheterization. In renal patients, the basilic vein use as an arteriovenous graft or fistula for haemodialysis access. During a routine dissection in the department of anatomy in Isfahan, we observed a variation in the left arm of an infant boy (six months old). The cephalic and basilic veins directly joined together in the middle of the cubital fossa. The brachial vein began from this point and, unlike the normal anatomy location, there was no paired brachial vein; rather, it was one unpaired brachial vein.

## Introduction

Understanding anatomic organ variation and its arterial and venous supply is useful for nurses and physicians, such as surgeons or radiologists.^1-4^ In clinics, most surface veins usage is related to the upper limbs. Usually in the upper limb, there are some major veins that pass a specific path. These veins consist of basilic, cephalic, and median cubital veins as the major superficial veins. Basilic and cephalic veins begin their path from around the wrist and continue towards the upper region of the forearm. The basilic vein becomes deep around the mid-arm, while the cephalic vein becomes deep around the upper forearm, in deltopectoral groove. The median cubital vein merges with different forms of these two veins in the elbow region. Usually, cephalic and basilic veins communicate with each other via a median cubital vein.^5-7^

Basilic vein is an appropriate vein for venepuncture. In renal patients, the basilic vein use as an arteriovenous graft or fistula for haemodialysis access.^8-12^

Javier and colleagues studied end-stage renal disease patients and divided all observed basilic variations into three types, while Arjmand et al reported six types of variation for the cephalic vein.^2,13-15^

In a study in Ahwaz, the cephalic vein variation was as follows: cephalic-median cephalic vein in 44.66%, cephalic-median cubital-median ante brachial in 30.1%, single-branched cephalic vein in 18.44%, cephalic-median cubital vein in 3.88%, and cephalic-median vein-basilic in 2.29%.^17^ In another study, the cephalic variation was investigated in 200 Korean subjects. The most common type featured a connection between the cephalic and basilica veins via the median cubital vein (50.1%).^18^

## Case Presentation

We observed unusual cephalic and basilic veins in the left arm of an infant boy (6 months old) that was dissected in a plastination laboratory in the Department of Anatomy of Isfahan University, in 2016.

**Figure 1:**
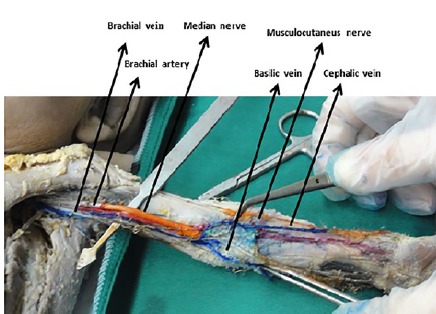
This figure shows cephalic and basilic vein directly joined together in the middle of cubital fossa and brachial vein started from this point.

No variation was observed in the right upper limb. In the left hand, the cephalic vein began in the roof of the anatomical snuffbox from the radial side of the venous network and continue in the lateral border of the limb. It then ascended in front of the elbow, superficial to a groove between the brachioradialis and biceps, crossing superficially to the lateral cutaneous nerve of the forearm. The basilic vein ran up the medial border of the limb. Then, in the middle of cubital fossa, the cephalic and basilic veins directly joined together and the brachial vein started from this point. Unlike the normal anatomical location, there was no paired brachial vein (Figure 1).

**Figure 2:**
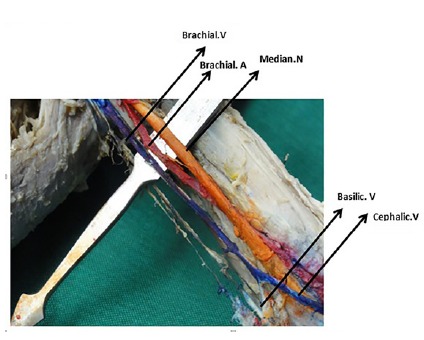
There was not paired brachial vein but a single brachial vein run up along the brachial artery.

The cephalic vein did not run up the normal passage in the left arm and in the lateral border of arm there was no continuation of the cephalic vein. The latter directly joined the basilic vein in the middle elbow region for formation of a brachial vein (Figure 2).

## Discussion

The superficial veins are most commonly used for vein puncture, transfusion, bypass graft, and cardiac catheterization. Kaiser and colleagues described a female cadaver with renal failure and preeclampsia with a basilic vein complication due to lack of information on basilic vein. She developed severe upper-extremity oedema, because the brachiobasilic connection in the cubital fossa.^3^ So, this report emphasizes the influence of venous anatomical variability on access outcomes. Several studies demonstrated many variations of cephalic and basilic veins, and knowing about these variations is important. As noted earlier, Anaya-Ayala et al classified basilic variations into three types: The type 1 anatomy was defined as being consistent with the description in most anatomical atlases. In the proximal third of the upper arm, near the axilla, the basilic connects the brachial venous system to create the axillary vein. In the types 2 and 3 anatomies, the basilic vein joins the brachial venous system in the middle or lower third of the upper brachium. In type 2, the brachial veins are double at the level of the brachiobasilic junction. In the type 3 anatomy, there is only a brachial vein above the level of convergence with the basilic vein (2).

Kim et al reported that the external jugular and cephalic veins were connect together to form a common trunk. The cephalic vein derange into the internal jugular vein.^16,19^

In the present study, we tried to present a cephalic and basilic vein variation in the upper limb, similar to type 3. Moreover, the cephalic vein did not run up the normal passage in the arm and it joined the basilic vein in the middle elbow region to form a brachial vein like type 4. Information about this variation might be considered by the operator when using an arteriovenous fistula for haemodialysis in cases with renal disease.

In a study in Ahwaz, the cephalic vein variation was as follows: cephalic-median cephalic vein in 44.66%, cephalic-median cubital-median ante brachial in 30.1%, single-branched cephalic vein in 18.44%, cephalic-median cubital vein in 3.88%, and cephalic-median vein-basilic in 2.29%.^17^ In another study, the cephalic variation was investigated in 200 Korean subjects. The most common type featured a connection between the cephalic and basilica veins via the median cubital vein (50.1%).^18^

We searched the literature and found a report covering the different variations in cephalic and basilic veins, but we did not find any report of variations in the cephalic and basilic veins together that was similar to this case. The knowledge of the variations of the basilic and cephalic veins, may decrease the incidence of complications.

## Competing interests

None.

## Ethical approval

The study was approved by the Local Ethics Committee.

## Acknowledgements

This research was supported by Isfahan University of Medical Sciences.
